# Co-Occurrence of Arthritis and Stroke amongst Middle-Aged and Older Adults in Canada

**DOI:** 10.1155/2014/651921

**Published:** 2014-04-16

**Authors:** Roman Matveev, Chris I. Ardern

**Affiliations:** School of Kinesiology and Health Science, York University, Toronto, ON, Canada M3J 1P3

## Abstract

Arthritis is a chronic inflammatory condition commonly associated with mobility restriction and reduced activity. To date, the extent to which arthritis is an independent risk factor for stroke is unclear, and important, in light of an aging population. The purpose of this study was to (i) quantify the cross-sectional association between stroke and arthritis and (ii) to determine whether the relationship differed in physically active and inactivemiddle-aged and older adults. Data was derived from the 2010 Canadian Community Health Survey (*N* = 47 188; ≥30 y). Multivariable logistic regression was used to estimate the association between arthritis and stroke in models adjusted for age, physical activity (PA), and demographic factors. Overall, individuals with arthritis were 4 times more likely to report a history of stroke (OR = 3.8, 95% CI = 3.06–4.68), whereas those who were engaged in at least moderate PA (≥ 1.5 kcal/kg/day) were less than half as likely (0.45, 0.92−0.62). This effect was moderated by age, as younger (30–65 y: 3.27, 2.22–4.83) but not older adults (>65 y: 1.04, 0.8–1.35) with arthritis had elevated odds of stroke. Both physical inactivity and arthritis are associated with higher odds of stroke, effects of which are the strongest amongst 30–65 year olds.

## 1. Introduction

Stroke is a serious medical emergency and is the third leading cause of death in Canada [1]. Amongst the many known stroke risk factors, the INTERSTROKE study [2] identified 10 factors (including hypertension, smoking, alcohol intake, physical activity, diabetes, and cardiac causes) that are associated with 90% of all cases. While the stroke literature is extensive, few studies have examined the relationship of stroke to chronic diseases such as arthritis.

Current estimates project that nearly 4 million Canadians are living with arthritis and that, by 2026, this number is expected to increase to over 6 million [3]. Subsequently, the direct and indirect healthcare burden of arthritis is considerable, particularly when estimates of work-related disability are included [4]. Given that older adults represent a large and growing proportion of the Canadian population [5], the issue of complex chronicity, such as the co-occurrence and interrelation of conditions such as arthritis and stroke, must be given greater attention.

To date, research on the relationship between stroke and arthritis has been largely derived from studies of cardiovascular mortality [6]. A recent meta-analysis by Meune et al. [7] found that stroke was more commonly seen amongst individuals with rheumatoid arthritis (RA) (OR = 1.91, 95% CI = 1.73–2.12). In another study of RA patients in Glasgow, a trend towards a higher prevalence of stroke was also observed (*P* = 0.08) [8]. While a chronic inflammatory state may in part provide a mechanism by which, to support the arthritis-stroke link, specific arthritis treatments (procedures, surgeries, and medications) and a cluster of other risk factors (including disease mismanagement of lack of adequate CVD preventative care) are likely to contribute [9]. Because physical activity (PA) is associated with a lower risk of stroke [10], it stands to reason that PA may also play a key role in the co-occurrence of both arthritis and stroke in aging populations.

Therefore, the purpose of this study was (i) to quantify the association between arthritis and stroke, and (ii) to determine whether this relationship was attenuated once physical activity levels were taken into account.

## 2. Materials and Methods

### 2.1. Data Source

Data for this study was drawn from the 2010 Canadian Community Health Survey (CCHS), a cross-sectional survey that collects information on the health status, health care utilization, and health determinants of the Canadian population. About 65 000 individuals respond to the survey on an annual basis, representing all 117 health regions of Canada [11].

### 2.2. Survey Methods

#### 2.2.1. Variable Analysis

Of the initial 62 909 respondents, participants less than age 30 y (*N* = 15 721), and those with missing data for any of the study variables, were excluded from this study, leaving a final analytic sample of 47 617. The stroke prevalence variable was derived from individual responses to the survey question “Suffers from effects of stroke.” Due to a lack of available stroke-specific information (i.e., stroke subtypes) and to avoid additional power loss, all stroke types were collapsed into an overall stroke variable. Out of the 47 617, there were 980 individuals with stroke. Of those who were ≥65 y, 683 suffered from stroke (4.5%).

Self-reported (“yes”/“no”) comorbid conditions included history of arthritis, diabetes (type 1, 2, and gestational), hypertension, and heart disease. Individuals who were unsure about whether or not they had these conditions were excluded from the final analysis (*N* = 5 234). Weight (kg) and height (m) were self-reported and used to calculate body mass index (BMI). BMI was subsequently used to classify participants as “normal weight” (<24.9 kg/m^2^), “overweight” (25.0–29.9 kg/m^2^), and “obese” (≥30 kg/m^2^).

Age, sex, cigarette smoking, and alcohol consumption were included as covariates in all analyses. Physical activity was calculated on the basis of self-reported leisure and transportation-related activities and classified as “active” (≥3.0 kcal/kg/day), “moderately active” (1.5–2.9 kcal/kg/day), and “inactive” (<1.5 kcal/kg/day).

#### 2.2.2. Statistical Analysis

Characteristics of the sample (with and without stroke) are presented as frequencies (%) and compared by chi-square analysis. Bivariate logistic regression was subsequently used to examine the association between stroke and arthritis with other stroke-related risk factors. A backwards stepwise logistic regression (with entry at *P* < 0.10) was then used to examine the effect of each risk factor, stratified by physical activity (referent = “no” risk factor group within each activity category). SAS 9.3 was used for all analyses, with statistical significance set at alpha <0.05. All analyses were weighted (using survey procedures) to ensure representativeness to the Canadian population.

## 3. Results

Characteristics of the sample are presented in Table 1, stratified by stroke history. After accounting for missing cases and outliers, the final analytical sample of 47 617 contained 980 individuals with stroke and 13 126 with arthritis. Approximately half of individuals with stroke also had arthritis. Compared to the individuals who did not suffer from stroke, individuals with stroke were more likely to have at least one cardiometabolic risk factor.

Table 2 presents the unadjusted logistic regression between stroke and arthritis. Individuals with arthritis were almost four times more likely to have a history of stroke (odds ratio: 3.8, 95% confidence interval: 3.06–4.68). They were also much more likely to have hypertension, heart disease, and diabetes, be more than 65 y old, be a former smoker, and be either underweight or obese. Compared to inactive men and women (OR = 1.00, referent), being physically active (0.45, 0.32–0.62) or moderately active (0.56, 0.43–0.72) was associated with lower odds of stroke as was regular or occasional consumption of alcohol (0.36, 0.29–0.46).

Interactions between age, sex, and physical activity with arthritis and stroke were explored and revealed a significant age-by-arthritis effect. In age-stratified analyses (Table 3), differences in stroke-related risk factors emerged. For example, arthritis was significantly associated with stroke amongst the younger (30–65 y: 3.27, 2.22–4.83) but not older age groups, whereas hypertension, alcohol consumption, and heart disease all remained significantly associated with stroke. By contrast, diabetes, male sex, and physical activity levels were not related to stroke within the subset of older adults.

**Figure 1:**
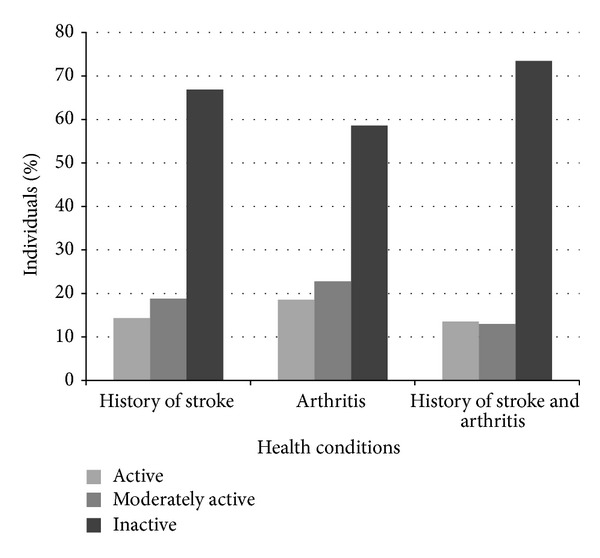
Physical activity levels within CCHS participants with stroke, arthritis, and both conditions together.

**Figure 2:**
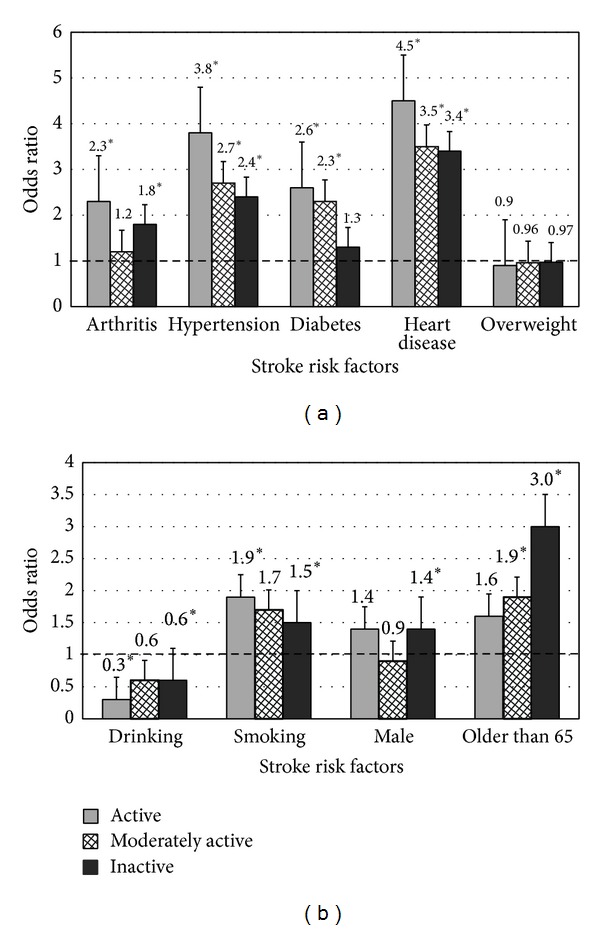
Odds of stroke-related risk factors according to physical activity level. ∗ denotes significance at *P* < 0.05. Referent = individuals within each physical activity category without the risk factor of interest.

The association between stroke, arthritis, and physical activity level is presented in Figure 1. As expected, the prevalence of inactivity was found to be the highest amongst participants with a history of stroke and arthritis (73% inactive).  Figure 2 presents the joint association between selected nonmodifiable (age, male sex) and modifiable risk factors (arthritis, hypertension, diabetes, heart disease, alcohol consumption, cigarette smoking, and overweight/obese) and physical activity level (inactive, moderately-active, and active) groups. After adjusting for covariates, arthritis was associated with an elevated odds of stroke amongst both active (2.18, 1.45–3.26) and inactive (1.50, 1.24–1.82) individuals (Figure 2).

## 4. Discussion

Prevalence of stroke in this sample (2.1%) is similar to other population-based estimates in Canada [12, 13], and reflect age-, and sex-based variations seen in the US [14]. Results from this analysis reveal that arthritis is associated with a greater likelihood of stroke, even after adjusting for traditional risk factors. Moreover, these findings suggest that this association may be the greatest within the young-to-middle aged (30–65 y) population, wherein the protective effect of PA may be most pronounced. As a consequence, targeting younger individuals with chronic conditions such as arthritis may be beneficial for reducing future stroke prevalence.

While the relationship between stroke and risk factors, such as hypertension, heart disease, and diabetes, are well known, research on the arthritis-stroke relationship has yielded mixed results. For example, in a study of RA patients, Nadareishvili et al. [15] found that the odds of any stroke were 1.64 (1.16–2.30) and 2.66 (1.24–5.70) for ischemic stroke. Similarly, in a large study by Solomon et al.  [16], patients with RA had approximately double the rate of myocardial infarction and stroke, whereas a population-based study by Bacani et al. [17] found no difference in the rates of cerebrovascular disease in RA (versus non-RA) participants. Consistent with our findings, a register-based cohort study in Denmark has also reported a stronger association between arthritis and stroke in younger (versus older) adults [18].

At present, it is not fully understood why individuals with arthritis may report higher likelihood of stroke. Rincón et al. [19] have speculated that a state of chronic inflammation may contribute to an elevated risk of stroke, whereas others have proposed that the pathway may be more related to atrial fibrillation and heart failure [20], either directly [21] or through long-term use of glucocorticoids [22] and NSAID medication [23]. These studies notwithstanding our analyses revealed an association with most of the traditional risk factors for stroke (e.g., hypertension, heart disease, and diabetes). According to the American Heart Association [24], approximately 70% of all men who develop high blood pressure in middle age will experience a cardiovascular disease event, including stroke, by age 85. Sacco [25] has found that hypertension, cardiac disease, smoking, alcohol abuse, and physical inactivity have all been identified as predictors of late stroke occurrence. Although leisure-time PA is associated with a lower risk of ischemic (and any) stroke [26], arthritis may be an additional (mobility and pain-related) barrier for PA [27]. It is therefore not surprising that 73% of individuals in our sample with arthritis and stroke were physically inactive. In our age-adjusted model, being overweight, obese, or underweight was associated with higher odds of stroke only amongst young-to-middle aged individuals. Interestingly, overweight/obesity was somewhat protective of stroke, and may be explained in part by its impact on other chronic disease risk factors that may partially mediate the obesity-stroke relationship [28].

## 5. Limitations

As with any secondary analysis of population-level data, a number of important caveats are warranted. First, since CCHS relies on self-report of health behaviour and medical history information, we cannot exclude the possibility of recall and healthy responder bias. Second, stroke was assessed according to a single-item question “Do you suffer from effects of stroke” which potentially conflates the stroke event with its medical sequelae and could contribute to reporting errors. In addition, it was not possible to differentiate the arthritis and stroke subtypes and severity, which in turn, may have contributed to an under- or overestimation of the overall relationship and is an area in need of additional work. Moreover, the relationship between arthritis and stroke subtype may vary. For example, gout may be related to ischemic and hemorrhagic stroke through increases in serum uric acid levels [29, 30], whereas RA is associated with elevated odds of atherosclerotic CVD [31] and subarachnoid haemorrhage [32]. Finally, use of different types of arthritis medications, such as NSAIDs, can also increase the risk of stroke [33, 34]. Unfortunately, this relationship could not be verified because no medication information was available in the CCHS.

## 6. Conclusions 

Results from this study suggest that arthritis is associated with higher odds of stroke even after adjusting for covariates. Within the context of an aging population, the finding of fourfold higher odds of stroke amongst those with arthritis provides important perspective for targeted interventions that support the prevention and management of chronic disease. Given current trends in aging and arthritis, further research is necessary to assess the long-term relationship between arthritis and stroke and to provide supports for healthful PA within this segment of older adults in Canada.

## Figures and Tables

**Table 1:** Baseline variables compared by stroke prevalence.

	History of Stroke	No History of Stroke	Weighted *P*-value
Gender (%)			
Female	514 (1.9)	26076 (98.1)	NS
Male	469 (2.2)	20662 (97.8)	
Age (years) (%)			
30–65	300 (0.9)	31684 (99.1)	<0.0001
>65	683 (4.3)	15054 (95.7)	
Arthritis (%)			
Yes	511 (3.9)	12615 (96.1)	<0.001
No	469 (1.4)	34022 (98.6)	
Hypertension (%)			
Yes	605 (4.4)	13273 (95.6)	<0.001
No	374 (1.1)	33369 (98.9)	
Heart Disease (%)			
Yes	379 (8.8)	3940 (91.2)	<0.001
No	590 (1.4)	42691 (98.6)	
Diabetes (%)			
Yes	268 (5.4)	4715 (94.6)	<0.001
No	713 (1.7)	41995 (98.3)	
Alcohol (%)			
No Drinks Past 12 Months	416 (3.9)	10375 (96.1)	<0.001
Regular or Occasional Drinker	544 (1.5)	35669 (98.5)	
BMI Class (%)			
Normal (<24.9 kg/m^2^)	463 (2.2)	20819 (97.8)	<0.1
Overweight (25.0–29.9 kg/m^2^)	290 (1.7)	16326 (98.3)	
Obese (>30.0 kg/m^2^)	230 (2.3)	9593 (97.7)	
Smoking Status (%)			
Never Smoked	274 (1.8)	15060 (98.2)	0.05
Smokes Daily or Occasionally	188 (1.9)	9742 (98.1)	
Former Smoker	511 (2.3)	21670 (97.7)	
Physical Activity			
Inactive (<1.5 kcal/kg/day)	579 (2.4)	23117 (97.6)	<0.001
Active (≥3.0 kcal/kg/day)	119 (1.1)	10868 (98.9)	
Moderately Active (1.5–2.9 kkd)	180 (1.5)	11682 (98.5)	

**Table 2:** Bivariate relationship between stroke risk factors.

	Odds Ratio	95% CI
Sex		
Female	1.00	Ref
Male	1.06	0.85–1.32
Age (years)		
30–65	1.00	Ref
>65	5.74	4.55–7.23
Arthritis		
No	1.00	Ref
Yes	3.78	3.06–4.68
Hypertension		
No	1.00	Ref
Yes	5.78	4.64–7.20
Heart Disease		
No	1.00	Ref
Yes	9.83	7.78–12.41
Diabetes		
No	1.00	Ref
Yes	3.88	3.03–4.98
Alcohol		
No Drinks Past 12 Months	1.00	Ref
Regular or Occasional Drinker	0.36	0.29–0.46
BMI		
Normal (<24.9 kg/m^2^)	1.00 (REF)	
Overweight (25.0–29.9 kg/m^2^)	0.97	0.76–1.24
Obese (>30.0 kg/m^2^)	1.31	0.99–1.73
Smoking Status		
Never Smoked	1.00 (REF)	
Smokes Daily or Occasionally	1.54	1.10–2.15
Former Smoker	1.44	1.14–1.83
Physical Activity		
Inactive (<1.5 kcal/kg/day)	1.00 (REF)	
Moderately Active (1.5–2.9 kcal/kg/day)	0.45	0.32–0.62
Active (≥3.0 kcal/kg/day)	0.56	0.43–0.72

**Table 3:** Odds of stroke in middle-aged and older adults.

	Middle-Aged Adults (30–65 y)		Older Adults (>65 y)	
	Odds Ratio*	95% CI	Odds Ratio*	95% CI
Male Sex	0.90	0.61–1.33	1.52	1.13–2.04
Arthritis	3.27	2.22–4.83	1.06	0.82–1.38
Hypertension	4.05	2.65–6.19	1.89	1.42–2.52
Diabetes	2.81	1.76–4.49	1.11	0.79–1.56
Heart Disease	3.58	2.24–5.71	3.33	2.54–4.36
Alcohol (Regular or Occasional Drinker)	0.33	0.22–0.50	0.74	0.56–0.97
BMI (kg/m^2^)	0.93	0.90–0.97	0.98	0.95–1.01

*Stepwise logistic regression considering all risk factors listed.

## References

[1] http://www.statcan.gc.ca/pub/84f0209x/84f0209x2008000-eng.pdf.

[2] O’Donnell MJ, Xavier D, Liu L (2010). Risk factors for ischaemic and intracerebral haemorrhagic stroke in 22 countries (the INTERSTROKE study): a case-control study. *The Lancet*.

[3] Lagacé C, Perruccio A, DesMeules M, Badley E, Badley EM, DesMeules M (2003). The impact of arthritis on canadians. *Health Canada. Arthritis in Canada: An Ongoing Challenge*.

[4] Zhang W, Anis AH (2011). The economic burden of rheumatoid arthritis: beyond health care costs. *Clinical Rheumatology*.

[5] O’Donnell S, Lagacé C, McRae L, Bancej C (2011). Life with arthritis in canada: a personal and public health challenge. *Chronic Diseases in Canada*.

[6] Lévy L, Fautrel B, Barnetche T, Schaeverbeke T (2008). Incidence and risk of fatal myocardial infarction and stroke events in rheumatoid arthritis patients. A systematic review of the literature. *Clinical and Experimental Rheumatology*.

[7] Meune C, Touzé E, Trinquart L, Allanore Y (2010). High risk of clinical cardiovascular events in rheumatoid arthritis: levels of associations of myocardial infarction and stroke through a systematic review and meta-analysis. *Archives of Cardiovascular Diseases*.

[8] McEntegart A, Capell HA, Creran D, Rumley A, Woodward M, Lowe GDO (2001). Cardiovascular risk factors, including thrombotic variables, in a population with rheumatoid arthritis. *Rheumatology*.

[9] Solomon DH, Karlson EW, Rimm EB (2003). Cardiovascular morbidity and mortality in women diagnosed with rheumatoid arthritis. *Circulation*.

[10] Lee CD, Folsom AR, Blair SN (2003). Physical activity and stroke risk: a meta-analysis. *Stroke*.

[11] http://www23.statcan.gc.ca/imdb/p2SV.pl?Function=getSurvey&SDDS=3226.

[12] http://www.phac-aspc.gc.ca/publicat/2009/cvd-avc/pdf/cvd-avs-2009-eng.pdf.

[13] Hollander M, Koudstaal PJ, Bots ML, Grobbee DE, Hofman A, Breteler MMB (2003). Incidence, risk, and case fatality of first ever stroke in the elderly population. The Rotterdam Study. *Journal of Neurology Neurosurgery and Psychiatry*.

[14] Falcone G, Chong JY (2007). Gender differences in stroke among older adults. *Geriatrics and Aging*.

[15] Nadareishvili Z, Michaud K, Hallenbeck JM, Wolfe F (2008). Cardiovascular, rheumatologic, and pharmacologic predictors of stroke in patients with rheumatoid arthritis: a nested, case-control study. *Arthritis Care and Research*.

[16] Solomon DH, Goodson NJ, Katz JN (2006). Patterns of cardiovascular risk in rheumatoid arthritis. *Annals of the Rheumatic Diseases*.

[17] Bacani AK, Gabriel SE, Crowson CS, Heit JA, Matteson EL (2012). Noncardiac vascular disease in rheumatoid arthritis: increase in venous thromboembolic events?. *Arthritis and Rheumatism*.

[18] Lindhardsen J, Ahlehoff O, Gislason GH (2012). Risk of atrial fibrillation and stroke in rheumatoid arthritis: danish nationwide cohort study. *British Medical Journal*.

[19] Rincón I, Williams K, Stern MP, Freeman GL, O’Leary DH, Escalantel A (2003). Association between carotid atherosclerosis and markers of inflammation in rheumatoid arthritis patients and healthy subjects. *Arthritis and Rheumatism*.

[20] Wolfe F, Michaud K (2004). Heart failure in rheumatoid arthritis: rates, predictors, and the effect of anti-tumor necrosis factor therapy. *American Journal of Medicine*.

[21] Roumie CL, Mitchel EF, Kaltenbach L, Arbogast PG, Gideon P, Griffin MR (2008). Nonaspirin NSAIDs, cyclooxygenase 2 inhibitors, and the risk for stroke. *Stroke*.

[22] Christiansen CF, Christensen S, Mehnert F, Cummings SR, Chapurlat RD, Sørensen HT (2009). Glucocorticoid use and risk of atrial fibrillation or flutter: a population-based, case-control study. *Archives of Internal Medicine*.

[23] Chang C-H, Shau W-Y, Kuo C-W, Chen S-T, Lai M-S (2010). Increased risk of stroke associated with nonsteroidal anti-inflammatory drugs: a nationwide case-crossover study. *Stroke*.

[24] http://www.sciencedaily.com/releases/2011/12/111219203849.htm.

[25] Sacco RL (1998). Identifying patient populations at high risk for stroke. *Neurology*.

[26] Hu G, Sarti C, Jousilahti P, Silventoinen K, Barengo NC, Tuomilehto J (2005). Leisure time, occupational, and commuting physical activity and the risk of stroke. *Stroke*.

[27] Bolen J, Murphy L, Greenlund K (2009). Arthritis as a potential barrier to physical activity among adults with heart disease—United States, 2005 and 2007. *Morbidity and Mortality Weekly Report*.

[28] Kurth T, Gaziano JM, Berger K (2002). Body mass index and the risk of stroke in men. *Archives of Internal Medicine*.

[29] Chen J-H, Chuang S-Y, Chen H-J, Wen-Ting YEH, Wen-Harn PAN (2009). Serum uric acid level as an independent risk factor for all-cause, cardiovascular, and ischemic stroke mortality: a chinese cohort study. *Arthritis Care and Research*.

[30] Weir CJ, Muir SW, Walters MR, Lees KR (2003). Serum urate as an independent predictor of poor outcome and future vascular events after acute stroke. *Stroke*.

[31] Aubry M-C, Maradit-Kremers H, Reinalda MS, Crowson CS, Edwards WD, Gabriel SE (2007). Differences in atherosclerotic coronary heart disease between subjects with and without rheumatoid arthritis. *Journal of Rheumatology*.

[32] Ramagopalan SV, Wotton CJ, Handel AE, Yeates D, Goldacre MJ (2011). Risk of subarachnoid haemorrhage in people admitted to hospital with selected immune-mediated diseases: record-linkage studies. *BMC Neurology*.

[33] Taubert KA (2008). Can patients with cardiovascular disease take nonsteroidal antiinflammatory drugs?. *Circulation*.

[34] Madhok R, Wu O, McKellar G, Singh G (2006). Non-steroidal anti-inflammatory drugs—changes in prescribing may be warranted. *Rheumatology*.

